# Supplementary material for: Screening for Low Energy Availability in Male Athletes: Attempted Validation of LEAM-Q
Source: Nutrients. 2022 Apr 29;14(9):1873. doi: 10.3390/nu14091873 (PMC9101736; doi:10.3390/nu14091873)
Supplement: Supplementary file 1 [file nutrients-14-01873-s001.zip › Supplement File S1 LEAM Q scoring complete version.pdf]

# LEAM Q -

# A questionnaire for male athletes

Contacts:

Anna Melin, PhD, Associate Professor, MSc clinical nutrition, registered dietitian
Department of Sport Science, Faulty of Social Sciences, Linneaus University, Sweden
email: anna.melin@lnu.se

Monica K. Torstveit, PhD, Associate Professor, exercise scientist
University of Agder, Faculty of Health- and Sport Sciences, Kristiansand, Norway
email: monica.k.torstveit@uia.no

Louise M. Burke, PhD, Professorial Fellow, Accredited Practising Dietitian
Exercise and Nutrition Research Program, Mary MacKillop Institute for Health Research
Australian Catholic University, Australia
Email: louise.burke@acu.edu.au

**1 A: Do you feel dizzy when you rise quickly?**
3 Yes, several times a day, 2 Yes, several times a week, 1 Yes, once or twice a week or more seldom 0 Rarely or never
**1 B: Do you experience problems with vision (blurring, seeing spots, tunnel vision, etc.)**
3 Yes, several times a day 2 Yes, several times a week 1 Yes, once or twice a week or more seldom 0 Rarely or never
**2 A: Do you feel gaseous or bloated in the abdomen?**
3 Yes, several times a day, 2 Yes, several times a week, 1 Yes, once or twice a week or more seldom 0 Rarely or never
**2 B: Do you get cramps or stomach ache?**
3 Yes, several times a day, 2 Yes, several times a week, 1 Yes, once or twice a week or more seldom 0 Rarely or never
**2 C: How often d0 you have bowel movements on average?**
1 Several times a day, 0 once a day, 2 Every second day, 3 Twice a week, 4 Once a week or more rarely
2 D: **How would you describe your normal stool?**
0 Normal (soft), 1 Diarrhoea-like (watery), 2 Hard and dry
**3 A: Are you very cold even when you are normally dressed?**
3 Yes, almost every day, 2 Several times a week, 1 Once or twice a week or more seldom, 0 Rarely or never
**3B: Do you dress more warmly than your companions regardless of the weather?** 3 yes, almost always 1 Yes, sometimes 0 rarely or never
**4 A: How many acute injuries have you had during the past 6 months?**
The number of acute injuries is the score
**4 B: How many overload injuries (the same reoccurring overload injury, counts as a new injury for every new period) have you had during the past 6 months?**
The number of overload injuries is the score
**4 C. How many pauses in training have you had due to illness during the past months?**
The number of pauses in training due to illness is the score
**4 D. During the last 6 months, how many days in a row, at the most, have you been absent from training/competition or not been able to perform optimally at training/competition due to an injury (acute/overload) or illness?**

| | Non | 1-7 days | 8-14 days | 15-21 days | More than 22 days |
|---|---|---|---|---|---|
| Acute injury | 0 | 1 | 2 | 3 | 4 |
| Overload injury | 0 | 1 | 2 | 3 | 4 |
| Illness | 0 | 1 | 2 | 3 | 4 |

**5 A:1 I feel tired from work/school**
3 Yes, several times a day, 2 Yes, several times a week, 1 Yes, once or twice a week or more seldom, 0 Rarely or never
**5 A:2 I feel overtired**
3 Yes, several times a day, 2 Yes, several times a week, 1 Yes, once or twice a week or more seldom 0 Rarely or never

**5 A:3 I'm unable to concentrate well**
3 Yes, several times a day, 2 Yes, several times a week, 1 Yes, once or twice a week or more seldom,
0 Rarely or never
**5 A:4 I feel lethargic**
3 Yes, several times a day, 2 Yes, several times a week, 1 Yes, once or twice a week or more seldom,
0 Rarely or never
**5 A:5 I put off making decisions**
3 Yes, always 2 Yes, often 1 Yes, sometimes 0 Rarely or never
**5 B:1 Parts of my body are aching**
3 Yes, several times a day, 2 Yes, several times a week, 1 Yes, once or twice a week or more seldom 0 Rarely or never
**5 B:2 My muscles feels stiff or tense during training**
3 Yes, almost every training session, 2 Yes, often, 1 Yes, sometimes, 0 Rarely or never
**5 B:3 I have muscle pain after performance**
3 Yes, after almost every training session, 2 Yes, often, 1 Yes, sometimes, 0 Rarely or never
**5 B:4 I feel vulnerable to injuries**
3 Yes, always, 2 Yes, in most training periods, 1 Yes, in some training periods, 0 Rarely or never
**5 B:5 I have a headache**
3 Yes, almost daily, 2 Yes, several days a week, 1 Yes, once or twice a week or more seldom, 0 Rarely or never
**5 B:6 I feel physically exhausted**
3 Yes, almost daily, 2 Yes, several days a week, 1 Yes, once or twice a week or more seldom, 0 Rarely or never
**5 B:7 I feel strong and am making good progress with my strength training**
0 Yes, always 1 Yes, in most training periods 2 Yes, in some training periods 3 Rarely or never
**5 C:1 I get enough sleep**
0 Yes, almost every night, 1 Yes, several nights a week, 2 Yes, once or twice a week or more seldom, 3 Rarely or never
**5 C:2 I fall asleep satisfied and relaxed**
0 Yes, almost every night, 1 Yes, several nights a week, 2 Yes, once or twice a week or more seldom, 3 Rarely or never
**5 C:3 I wake up and well rested**
0 Yes, almost every morning, 1 Yes, several days a week, 2 Yes, once or twice a week or more seldom 3 Rarely or never
**5 C:4 I sleep restlessly**
3 Yes, almost every night, 2 Yes, several nights a week, 1 Yes, once or twice a week or more seldom 0 Rarely or never
**5 C:5 My sleep is easily interrupted**
3 Yes, almost every night, 2 Yes, several nights a week, 1 Yes, once or twice a week or more seldom 0 Rarely or never
**5 D:1 I recover well physically**
0 Yes, after almost all training sessions, 1 Yes, often, 2 Yes, sometimes, 3 Rarely or never
**5 D:2 I'm in good physical shape**
0 Yes, always, 1 Yes, mostly, 2 Yes, sometimes, 3 Rarely or never

**5 D:3 I feel I am achieving the progress in training and competition that I deserve**
0 Yes, always, 1 Yes, in most training periods, 2 Yes, in some training periods, 3 Rarely or never
**5 D:4 My body feel strong**
0 Yes, almost every day, 1 Yes, several days a week, 2 Yes, once or twice a week or more seldom, 3 Rarely or never
**5 E:1 I feel very energetic in general**
0 Yes, almost every day, 1 Yes, several days a week, 2 Yes, once or twice a week or more seldom, 3 Rarely or never
**5 E:2 I feel invigorated for training sessions and ready to perform well**
0 Yes, almost every day, 1 Yes, several days a week, 2 Yes, once or twice a week or more seldom, 3 Rarely or never
**5 E:3 I feel happy and on top of my life outside sport**
0 Yes, almost every day, 1 Yes, several days a week, 2 Yes, once or twice a week or more seldom, 3 Rarely or never
**5 E:4 I feel down and less happy than I used to feel or would like to feel**
3 Yes, almost every day, 2 Yes, several days a week, 1 Yes, once or twice a week or more seldom, 0 Rarely or never
**5 F:1a I would rate my sex drive as**
0 high, 1 moderate, 2 low, 3 I don't have much interest in sex
**5 F:1b over the last month I would rate my sex drive as**
0 stronger than usual, 0 about the same, 1 a little less than usual 2 much less than usual
**5 F:2a Morning erections: over the last month this has happened**
0 5-7 per week, 0 3-4 a week, 1 1-2 a week, 2 rarely or never
**5 F:2b compared to what you would consider normal for you is this**
0 more often, 0 about the same, 1 a little less often, 2 much less often
