# Supplementary material for: Screening for Low Energy Availability in Male Athletes: Attempted Validation of LEAM-Q
Source: Nutrients. 2022 Apr 29;14(9):1873. doi: 10.3390/nu14091873 (PMC9101736; doi:10.3390/nu14091873)
Supplement: Supplementary file 1 [file nutrients-14-01873-s001.zip › Supplement File S1 LEAM-Q complete version.pdf]

LEAM Q -

A questionnaire for male athletes

Contacts:

Anna Melin, PhD, Associate Professor, MSc clinical nutrition, registered dietitian
Department of Sport Science, Faulty of Social Sciences, Linneaus University, Sweden
email: anna.melin@lnu.se

Monica K. Torstveit, PhD, Associate Professor, exercise scientist
University of Agder, Faculty of Health- and Sport Sciences, Kristiansand, Norway
email: monica.k.torstveit@uia.no

Louise M. Burke, PhD, Professorial Fellow, Accredited Practising Dietitian
Exercise and Nutrition Research Program, Mary MacKillop Institute for Health Research
Australian Catholic University, Australia
Email: louise.burke@acu.edu.au

The low energy availability in males questionnaire (LEAM –Q), focuses on physiological symptoms of relative energy deficiency. The following pages contain questions regarding health, injuries, cold sensitivity, gastrointestinal function and recovery. We appreciate you taking the time to fill out the LEAM-Q and the results will be treated as confidential.

Name: ______________________

Address: ______________________

____

E-mail: ______________________

Cell phone: __________

Sport: ______________________

- How old were you when you began to specialize in your sport?:__________ age
- What level of athlete are you?
  Club ☐
  National team ☐
  Professional ☐
  Other ☐
- Are you a full time athlete? Yes ☐ No ☐
- If not, what occupation do you have beside your sport?
  Full time job ☐
  Part time job ☐
  Student ☐
  Other ☐
- What is your maximal oxygen consumption (Vo_2max)?

  ___________ml/kg/min or

  ____________l/min

  I do not know/I have never measured it ☐

- Your best results at World Championship, Olympic Games or World Cup?
  1st to 3rd place ☐
  4th to 6th place ☐
  7th to 10th place ☐
  11th place or lower ☐
  I have never competed at this level ☐
  I don't remember ☐

- Your normal amount of training in the preparation or basic period (not competition) on average per month:

  ____________________ hours/month

- Age: ____________(years)

- Height: ____________(cm)

- Present weight: ____________(kg)

- Your highest weight with your present height: ____________ (kg)

- Your lowest weight with your present height: ____________ (kg)

- What is your preferred body weight during competition? ____________ (kg)

- What is your body fat percentage (if it has been measured)? _______ (%)

- Chronic illness (e.g. diabetes, Crohn's Disease)?
  Yes ☐ No ☐

  If yes, which one (s)?

- Food allergy or intolerance (e.g. nut allergy, celiac disease, lactose intolerance)?
  Yes ☐ No ☐

  If yes, which one (s)?

---

1. Dizziness

Mark the response that most accurately describes your situation

A: Do you feel dizzy or lightheaded when you rise quickly?

☐ Yes, several times a day ☐ Yes, several times a week
☐ Yes, once or twice a week or more seldom ☐ Rarely or never

B: Do you experience problems with vision (blurring, seeing spots, tunnel vision, etc.)

☐ Yes, several times a day ☐ Yes, several times a week
☐ Yes, once or twice a week or more seldom ☐ Rarely or never

2. Gastrointestinal function

A: Do you feel gaseous or bloated in the abdomen?

☐ Yes, several times a day ☐ Yes, several times a week
☐ Yes, once or twice a week or more seldom ☐ Rarely or never

B: Do you get cramps or stomach ache?

☐ Yes, several times a day ☐ Yes, several times a week
☐ Yes, once or twice a week or more seldom ☐ Rarely or never

C: How often do you have bowel movements on average?

☐ Several times a day ☐ once a day ☐ Every second day ☐ Twice a week
☐ Once a week or more rarely

D: **How would you describe your normal stool?**

☐ Normal (soft) ☐ Diarrhoea-like (watery) ☐ Hard and dry

Comments regarding gastrointestinal function: ____________________

3. Regulation of body temperature at rest

A: Are **you very cold even when you are normally dressed?**

☐ Yes, almost every day ☐ Several times a week ☐ Once or twice a week or more seldom
☐ Rarely or never

B: Do you dress more warmly than your companions regardless of the weather?

☐ Yes, almost always ☐ Yes, sometimes ☐ Rarely or never

4. Health problem interfering with training or competition plans

Mark the response that most accurately describes your situation

In the following we will ask you some question regarding how often, during the last 6 month you have had to change plans concerning training or competition or not been able to perform your maximal during training due to a sport injury or illness. An *acute injury* appears suddenly for an obvious reason at a specific time (e.g. a sprain). An injury due to *overload* develops gradually (e.g. shin or Achilles, stress fracture).

A: How many acute injuries have you had during the past 6 months?

____________________ acute injuries.

B: How many overload injuries (the same reoccurring overload injury, counts as a new injury for every new period) have you had during the past 6 months?

____________________ overload injuries.

C. How many breaks in training have you had due to illness during the past 6 months?

____________________ breaks in training due to illness.

D. During the last 6 months, how many days in a row, at the most, have you been absent from training/competition or not been able to perform optimally at training/competition due to an injury (acute/overload) or illness?

	None	1-7 days	8-14 days	15-21 days	≥ 22 days
Acute injury	☐	☐	☐	☐	☐
Overload injury	☐	☐	☐	☐	☐
Illness	☐	☐	☐	☐	☐

Comments concerning your injuries: ____________________

____

____

Comments concerning your illnesses: ____________________

____

____

5. Well-being and recovery

Mark the response that most accurately describes your situation

A: Fatigue

A:1 I feel tired from work/school

☐ Yes, several times a day ☐ Yes, several times a week
☐ Yes, once or twice a week or more seldom ☐ Rarely or never

A:2 I feel overtired

☐ Yes, several times a day ☐ Yes, several times a week
☐ Yes, once or twice a week or more seldom ☐ Rarely or never

A:3 I'm unable to concentrate well

☐ Yes, several times a day ☐ Yes, several times a week
☐ Yes, once or twice a week or more seldom ☐ Rarely or never

A:4 I feel lethargic

☐ Yes, several times a day ☐ Yes, several times a week
☐ Yes, once or twice a week or more seldom ☐ Rarely or never

A:5 I put off making decisions

☐ Yes, always ☐ Yes, often ☐ Yes, sometimes ☐ rarely or never

B: Fitness

B:1 Parts of my body are aching

☐ Yes, several times a day ☐ Yes, several times a week
☐ Yes, once or twice a week or more seldom ☐ Rarely or never

B:2 My muscle feels stiff or tense during training

☐ Yes, almost every training session ☐ Yes, often ☐ Yes, sometimes ☐ Rarely or never

B:3 I have muscle pain after performance

☐ Yes, after almost every training session ☐ Yes, often ☐ Yes, sometimes ☐ Rarely or never

B:4 I feel vulnerable to injuries

☐ Yes, always ☐ Yes, in most training periods ☐ Yes, in some training periods ☐ Rarely or never

B:5 I have a headache

☐ Yes, almost daily ☐ Yes, several days a week ☐ Yes, once or twice a week or more seldom
☐ Rarely or never

B:6 I feel physically exhausted

☐ Yes, almost daily ☐ Yes, several days a week ☐ Yes, once or twice a week or more seldom
☐ Rarely or never

B:7 I feel strong and am making good progress with my strength training

☐ Yes, always ☐ Yes, in most training periods ☐ Yes, in some training periods ☐ Rarely or never

5. Continued

Mark the response that most accurately describes your situation

C: Sleep

C:1 I get enough sleep

☐ Yes, almost every night ☐ Yes, several nights a week ☐ Yes, once or twice a week or more seldom ☐ Rarely or never

C:2 I fall asleep satisfied and relaxed

☐ Yes, almost every night ☐ Yes, several nights a week ☐ Yes, once or twice a week or more seldom ☐ Rarely or never

C:3 I wake up well rested

☐ Yes, almost every morning ☐ Yes, several days a week ☐ Yes, once or twice a week or more seldom ☐ Rarely or never

C:4I sleep restlessly

☐ Yes, almost every night ☐ Yes, several nights a week ☐ Yes, once or twice a week or more seldom ☐ Rarely or never

C:5 My sleep is easily interrupted

☐ Yes, almost every night ☐ Yes, several nights a week ☐ Yes, once or twice a week or more seldom ☐ Rarely or never

C:6 During the last month, how many hours (mean/night) have you slept (this can be different from the number of hours that you have spent in bed) Sleep (hours) per night: ________________Hours

D: Recovery

D:1 I recover well physically

☐ Yes, after almost all training sessions ☐ Yes, often ☐ Yes, sometimes ☐ Rarely or never

D:2 I'm in good physical shape

☐ Yes, always ☐ Yes, mostly ☐ Yes, sometimes ☐ Rarely or never

D:3 I feel I'm achieving the progress in training and competition that I deserve

☐ Yes, always ☐ Yes, in most training periods ☐ Yes, in some training periods ☐ Rarely or never

D:4 My body feels strong

☐ Yes, almost every day ☐ Yes, several days a week ☐ Yes, once or twice a week or more seldom ☐ Rarely or never

Energy Levels

E:1 I feel very energetic in general

☐ Yes, almost every day ☐ Yes, several days a week ☐ Yes, once or twice a week or more seldom
☐ Rarely or never

E:2 I feel invigorated for training sessions and ready to perform well

☐ Yes, almost every day ☐ Yes, several days a week ☐ Yes, once or twice a week or more seldom
☐ Rarely or never

E-3 I feel happy and on top of my life outside sport

☐ Yes, almost every day ☐ Yes, several days a week ☐ Yes, once or twice a week or more seldom
☐ Rarely or never

E-4 I feel down and less happy than I used to feel or would like to feel

☐ Yes, almost every day ☐ Yes, several days a week ☐ Yes, once or twice a week or more seldom
☐ Rarely or never

Sex drive

F:1 Your sex drive can be a marker of the balance between training, rest and nutrition.

a) In general I would rate my sex drive as

☐ high ☐ moderate ☐ low ☐ I don't have much interest in sex

b) Over the last month I would rate my sex drive as

☐ stronger than usual ☐ about the same as usual ☐ a little less than usual
☐ much less than usual

F:2 It is common to wake in the morning with an erection

a) Over the last month, has this happened

☐ 5-7 per week ☐ 3-4 a week ☐ 1-2 a week ☐ Rarely or never

b) Compared to what you would consider is normal for you is this

☐ More often ☐ about the same ☐ a little less often ☐ much less often

Thank you!
