# Supplementary material for: Screening for Low Energy Availability in Male Athletes: Attempted Validation of LEAM-Q
Source: Nutrients. 2022 Apr 29;14(9):1873. doi: 10.3390/nu14091873 (PMC9101736; doi:10.3390/nu14091873)
Supplement: Supplementary file 1 [file nutrients-14-01873-s001.zip › Supplement File S2 LEAM-Q final.pdf]

Contacts:

Anna Melin, PhD, Associate Professor, MSc clinical nutrition, registered dietitian
Department of Sport Science, Faulty of Social Sciences, Linneaus University, Sweden
email: anna.melin@lnu.se

Monica K. Torstveit, PhD, Associate Professor, exercise scientist
University of Agder, Faculty of Health- and Sport Sciences, Kristiansand, Norway
email: monica.k.torstveit@uia.no

Louise M. Burke, PhD, Professorial Fellow, Accredited Practising Dietitian
Exercise and Nutrition Research Program, Mary MacKillop Institute for Health Research
Australian Catholic University, Australia
Email: louise.burke@acu.edu.au

The low energy availability in males questionnaire (LEAM –Q), focuses on physiological symptoms of relative energy deficiency. We appreciate you taking the time to fill out the LEAM-Q and the results will be treated as confidential.

Name: ______________________________

E-mail: ______________________________

Phone: ______________

Sport: ______________________________

- Age: __________(years)
- How old were you when you began to specialize in your sport?______________ Age
- Height: __________(cm)
- Present weight: __________(kg)
- Your highest weight with your present height: __________ (kg)
- Your lowest weight with your present height: __________ (kg)
- What is your preferred body weight during competition? __________ (kg)
- What is your body fat percentage (if it has been measured)? ______ (%)
- What level of athlete are you?
  Club ☐
  National team ☐
  Professional ☐
  Other ☐
- Are you a full-time athlete? Yes ☐ No ☐
- If not, what occupation do you have beside your sport?
  Full time job ☐
  Part time job ☐
  Student ☐
  Other ☐

- What is your maximal oxygen consumption (Vo_2max)?

  ___________ml/kg/min or ____________l/min

  I do not know/I have never measured it ☐

- Your best results at World Championship, Olympic Games or World Cup?
  1st to 3rd place ☐
  4th to 6th place ☐
  7th to 10th place ☐
  11th place or lower ☐
  I have never competed at this level ☐
  I don't remember ☐

- Your normal amount of training in the preparation or basic period (not competition) on average per week:

  __________________ hours/week

  Of this training time, roughly what percentage would you spend working at

  __________________ Low intensity (<35% VO_{2max})

  __________________ medium intensity (35-75% VO_{2max})

  __________________ high intensity (>70% VO_{2max})

- In your general life or work outside of prescribed training for your sport, would you describe your activity level as

  Low
  (low activity outside of formal training) ☐

  Medium
  (social sport, short commute) ☐

  High
  (physical job, long commute) ☐

Sex Drive

Mark the response that most accurately describes your situation

A: Your sex drive can be a marker of the balance between training, rest and nutrition.

1. In general I would rate my sex drive as
   ☐ high ☐ moderate ☐ low ☐ I don't have much interest in sex

2. Over the last month I would rate my sex drive as
   ☐ stronger than usual ☐ about the same as usual ☐ a little less than usual
   ☐ much less than usual

B: It is common to wake in the morning with an erection

1. Over the last month, has this happened
   ☐ 5-7 per week ☐ 3-4 a week ☐ 1-2 a week ☐ Rarely or never

Thank you!
