# Supplementary material for: Screening for Low Energy Availability in Male Athletes: Attempted Validation of LEAM-Q
Source: Nutrients. 2022 Apr 29;14(9):1873. doi: 10.3390/nu14091873 (PMC9101736; doi:10.3390/nu14091873)
Supplement: Supplementary file 1 [file nutrients-14-01873-s001.zip › Supplement File S2 LEAM-Q scoring key.pdf]

LEAM Q -

A questionnaire for male athletes

Contacts:

Anna Melin, PhD, Associate Professor, MSc clinical nutrition, registered dietitian
Department of Sport Science, Faulty of Social Sciences, Linneaus University, Sweden
email: anna.melin@lnu.se

Monica K. Torstveit, PhD, Associate Professor, exercise scientist
University of Agder, Faculty of Health- and Sport Sciences, Kristiansand, Norway
email: monica.k.torstveit@uia.no

Louise M. Burke, PhD, Professorial Fellow, Accredited Practising Dietitian
Exercise and Nutrition Research Program, Mary MacKillop Institute for Health Research
Australian Catholic University, Australia
Email: louise.burke@acu.edu.au

Sex Drive

A:1 I would rate my sex drive as

0 high, 1 moderate, 2 low, 3 I don't have much interest in sex

A:2 over the last month I would rate my sex drive as

0 stronger than usual, 0 about the same, 1 a little less than usual 2 much less than usual

B:1 Morning erections: over the last month this has happened

0 5-7 per week, 0 3-4 a week, 1 1-2 a week, 2 rarely or never

B:2 Compared to what you would consider normal for you is this

0 more often, 0 about the same, 1 a little less often, 2 much less often

Low sex drive is identified when
2 or more is scored on A1 OR
2 or more is scored on B1 AND 1 or more on B2
